# Left bundle branch area pacing: ready for prime time?

**DOI:** 10.1007/s12471-022-01681-z

**Published:** 2022-04-05

**Authors:** S. A. J. Timmer

**Affiliations:** Northwest Clinics/NWZ groep, Alkmaar, The Netherlands

In this issue of the *Netherlands Heart Journal*, Heckman and colleagues describe the feasibility and electrical properties of left bundle branch area pacing (LBBAP) [1]. In recent years, LBBAP has gained considerable attention worldwide as a pacing strategy in patients with atrioventricular block to reduce the risk of pacing-induced heart failure. LBBAP is a form of conduction system pacing (CSP) in which stimulation of the left bundle branch and/or left side of the interventricular septum with a permanent pacing lead maintains physiological electrical activation of the left ventricle [2]. LBBAP is a relatively new technique that was introduced to a wide audience by Huang et al. in 2019 and serves as an alternative to His bundle pacing (HBP) for preventing intraventricular dyssynchrony associated with traditional right ventricular pacing (RVP) [3]. In contrast to HBP, which targets a small zone in the membranous septum, the pacing lead in LBBAP is advanced through the septum well below the level of the tricuspid annulus, aiming at the bifurcation of His into the right and left bundle branches. This results in a larger target area, which is also less challenging to capture, seeing that the LBB is not encased in fibrous, non-conductive tissue as is the case with the bundle of His [4]. Therefore, LBBAP is perceived to be a more accessible/forgiving technique, suffering to a much lesser degree from potential drawbacks such as longer procedural time, higher pacing threshold, and R‑wave sensing issues associated with HBP [5, 6]. Both techniques are becoming increasingly popular in the Netherlands as well and especially LBBAP is experiencing exponential growth for the aforementioned reasons. Currently, CSP is being performed in approximately 20 Dutch clinics.

The majority of publications on LBBAP are international cohort studies and show high acute success rates for implantation, a low threshold during follow-up and surprisingly few complications, in line with the study by Heckman et al. [7]. In general, LBBAP results in fast and homogeneous activation of the left ventricle and is characterised by an rSR’ pattern in lead V1 together with a short left ventricular activation time (LVAT). More specifically, LBBAP is a collective term comprising various types of LV septal capture with and without direct stimulation of the specialised conduction system [8]. Interestingly, even without clear evidence of LBB capture a relatively short LVAT can be obtained by pacing the LV endocardium, albeit 10–15 ms longer [9]. This raises questions: Which type of capture is preferred and what LVAT is short enough to prevent pacing-induced cardiomyopathy? No prospective studies have been performed on this matter. Another important question is who will benefit most from LBBAP. Of note is that LBBAP in lieu of RVP for bradycardia is an add-on preventive technique from which the majority of patients will not benefit, because they would never develop pacing-induced cardiomyopathy in the first place. And if at the same time fluoroscopy times are longer and material costs are higher, this does not seem to justify an all-comer approach. Currently, the first randomised clinical trial comparing RVP with LBBAP is underway (LEAP; clinicaltrials.gov NCT04595487), hoping to answer some of these burning questions.

The most widely used pacing electrode for LBBAP is the 3830 (SelectSecure, Medtronic). This thin, lumen-less lead with a fixed helix design relies entirely on a guiding catheter for placement. The Medtronic C315 fixed curve workhorse sheath was primarily designed for HBP but can be easily employed for LBBAP as well. More recently, other medical device companies have introduced CSP tools (e.g. Biotronik Selectra 3D catheter, Boston Scientific Site Selective Pacing Catheters). These catheters have a larger diameter than the C315 sheath to facilitate passage of a traditional, stylet-driven pacing lead. The combination of a stiffer guiding sheath and stylet-driven pacing lead may provide better stability and forward push to penetrate the septum, which can occasionally be cumbersome with the Medtronic 3830 in areas with extensive scarring or fibrosis. Emerging data suggest comparable success rates, electrical properties and safety aspects to those of the Medtronic 3830 [10]. It should be noted that long-term follow-up data on lead survival for LBBAP are not available, although short-term data are reassuring. Nonetheless, the intraseptal fixation of the lead tip creates a hinge point with the lead body and will put additional mechanical strain on the conductors. Either way, the growing number of implanted 3830 leads will be accompanied by a larger number of patients requiring lead extraction. The deep intraseptal fixation together with the non-retractable helix design has raised concerns regarding extractability, although case reports suggest extraction with commercially available tools is feasible [11].

There is no doubt that LBBAP has great potential in the field of cardiac pacing. Nevertheless, many aspects need further refinement, and for LBBAP to reach prime time there is an urgent need for solid clinical evidence.
